# On the Effect of Mechanical Compression of the Head, as a Preventive and Cure in Certain Cases of Hydrocephalus

**Published:** 1821-10

**Authors:** G. Blane

**Affiliations:** Physician to the King.


### Original Communications, Select Observations, etc.

### *On the Effect of Mechanical Compression of the Head, as a Preventive and Cure in certain Cases of Hydrocephalus.* By Sir G. Blane, Bart. M.D. F.R.S. Physician to the King.

IN reflecting on the circumstances which characterize the history and description of hydrocephalus, some of the chief of which are, that it is very seldom met with but in very early life, and most commonly in infancy before the bregma is closed,—that there is in most cases a preternatural size of the head,—and that it is usually attended with a rachitic state of the bones and a general scrofulous flaccidity of the soft parts, and runs in particular families,—it occurred to me that the distention of the head and bregma is owing to a want of firmness and due resistance in the bony compages of the skull, which consequently yields to that effort of pressure with which the brain in its growth acts on its parietes. In reasoning further on the subject, it appeared to me conformable to some of the most approved principles of physiology, that, as there is a certain degree of tension and pressure necessary to the sound condition and action of parts, the withdrawing of this, by inviting afflux and congestion, produces serous effusion; and, for the like reason, there may be a deficiency of that interstitial absorption upon which the healthy state of this and all other parts of the living frame depends.

It was reflections of this kind, whether well or ill-founded, which some time ago suggested to me that mechanical compression of the head might be of use in the cure of hydrocephalus, and which induced me to make trial of it in a case which occurred to me about four months ago. A child, aged thirteen months, had a head of a preternatural size almost from birth, and the bregma was preternaturally large. The conformation of the child was otherwise defective; for there was a visible curvature of the spine, indicating a rachitic diathesis. He had for several months been subject to drowsiness; and latterly it was evident, from his screaming, and from raising his hand to his head, that there were occasional paroxysms of head-ach. There had also been for some time past a dilatation of the pupils. The functions of the bowels were not so much disordered as is generally the case in this disease, which was still in an unformed state.

I directed the head to be swathed with a roller, as tight as could be done without producing pain or uneasiness. The only other remedies were three leeches, applied once only to the temples and forehead, and a purge every two days of rhubarb and sulphate of potash: mercury was not used in any form. An immediate amendment took place, which continued so that all complaint was removed in less than three months, except the curvature of the spine; and he has continued well till now, that is, for eighteen months.

The only other case which has since occurred in my practice has been one which did not offer so fair a field for the trial. The subject was a child of three years of age, with a head preternaturally large, and the bregma not yet closed. No symptoms of hydrocephalus had appeared, but only a state of general delicacy. The swathing has apparently been of benefit, and may probably prevent a disorder naturally to be expected from such a conformation.

Before sitting down to write this, thinking it likely that a view of the subject seemingly so simple and obvious might have occurred to others, and deeming it possible that similar trials might have been made, and the practice exploded from being found unsuccessful, I consulted all the works I could meet with, but without finding any intimation of the theory or treatment here described. I therefore feel it my duty, even at this early period of my experience, to suggest the trial of it to others. I have elsewhere remarked, (Medico-Chir. Trans.) that one of the great purposes of the general and early diffusion of the valuable information contained in periodical works, is to offer suggestions of this nature, in order that they may undergo either a speedy confirmation or refutation, the experience of one individual being too limited to afford that satisfactory induction which medical truth demands, or too protracted for the decision of questions which, for the good of mankind, ought to be as prompt as possible.

It is necessary to caution practitioners against a too-active use of this method in recent and acute cases, where there is inflammatory affection, and therefore such a degree of tenderness as may make compression painful and prejudicial. Common discretion will suggest the times, circumstances, and degrees, in which this practice is admissible.

I would by no means rest the expediency of making further trials of this on the theoretical views which suggested it, but wish to refer entirely to experience. I am aware, indeed, that the theoretical views of others, being adverse to those which I have advanced, may produce a reluctance on the part of some practitioners to pay attention to what is here recommended. The cause of this disease has been referred by some to a depraved state of digestion, indicated by a distended abdomen and a vitiated state of the feculent matter, indicating a depraved action of the intestines, mesentery, and other chylopoietic viscera. But such disorders occur in innumerable instances without producing hydrocephalus; though it is easily conceivable how such a state of bowels may exasperate this disease, as well as all others of a rachitic or scrofulous nature, by producing depraved assimulation and scanty nutrition.

Other theorists are fond of representing hydrocephalus as proceeding, in most instances, from an inflammatory affection of the brain. There can be no doubt of such affection frequently taking place here; but I believe there are few instances of the acute state of hydrocephalus coming on without a predisposition, consisting in an enlarged head and bregma: and it has been plausibly maintained by some, that a relaxed state of vessels in particular parts is the main cause of inflammation. At all events, as it is found to run in families, this is a clear proof that it is a disease proceeding from a particular constitution, and not depending on accidental causes. One of the cases here related, having strong symptoms of predisposition, which were successfully combatted, strongly proves and illustrates this, and affords a useful practical hint to parents and medical attendants. I knew a family of the highest rank and respectability, who lost the whole of their young family by this disease, and died some years ago without heirs of their own body to their titles and estates. Some of these children would probably have been saved, had the preventive measures here stated been then known.

But, leaving all theoretical discussions out of the question, it is the author's purpose merely to submit what he has said as a suggestion to others who may be induced to make trial of it, and report the result to the public.

He has only further to suggest, that in case the utility of this practice should be confirmed by farther experience, it ought to be resorted to as a *preventive* as well as *curative* treatment, and applied whenever infants are perceived to have the head and bregma preternaturally large, without waiting its alarming manifestation by symptoms of hydrocephalus,—symptoms which, by this measure, may be happily averted.

P.S.—Since this article was first drawn up, (fourteen months ago,) several cases have occurred to myself and some of my friends, greatly in proof of the practice recommended; which, it ought to be understood, is not applicable to the acute state of the disorder, but either to those indications of predisposition consisting in a large head, a tardy closing of the bregma, broad pupils, and rachitic diathesis, or to cases in which the acute stage has been vanquished by evacuations and mercury, and where there is still a prospect of a fatal result, or of a protracted or imperfect cure. Both the subjects, whose cases have been detailed, have remained free from the disease, now eighteen months.

